# Intelligence

**Published:** 1820-06-01

**Authors:** 


### *Extract of a Letter from* George Williams, Esq.

*Uterine Hæmorrhage.* "After an easy labour, I delivered a delicate female of her sixth child, and the placenta followed in the usual time. Being called to another woman, and feeling the uterus distinctly in the right iliac region contracted to the size of a cricket ball, I left my patient, with strict injunctions that she should not be moved for some time, and when she was, to avoid the erect position as much possible.

"Two hours after this, I was alarmed by a call from my patient's husband, requesting my immediate attendance, as his wife was expiring; and hastened to her with all possible speed. She was in bed, with a countenance blanched and bloodless; the eyes sunk and half closed; the alæ nasi pinched in; in short, with a complete facies hippocratica. There was no pulsation in any of the tangible arteries, and all was apparently still in the region of the heart. A convulsive sob hove the chest about every half minute, and the pupils contracted on the approach of light; from no other circumstances, such was the state of asphyxia, could I determine that my patient lived!

"Applying my hand to the abdomen, I was astonished to find the uterus occupying as large a space in that cavity as it had done previous to delivery; this was an occurrence new to me, as I was not before aware, that the uterus would again dilate after so complete a contraction.

"Soon after I had quitted the house, the woman had been removed from the bed, and kept standing, whilst her linen was changed; during which period, several gushes of blood came from the uterus, which stopt spontaneously on the occurrence of syncope.

"The hæmorrhage, for the present, was stopped, but as the uterus continued dilated, there was every probability it would return with re-action, the patient, therefore, was by no means in safety.

"Previous to introducing my hand into the uterus, I thought it might materially assist to encircle the abdomen with a sheet doubled cornerwise, and giving each end of it to an assistant, directed them to pull as firm as possible; this was accordingly done. The convulsive throbbing had now given place to slow, but natural respiration; but still there was no pulse at the wrist.

"I poured a dose of tincture of opium and æther into the mouth, and to facilitate its descent to the stomach, passed my finger into the fauces; they contracted vigorously upon it; I irritated still more by moving my finger about, and produced an *effort to vomit.* The vomiting continued; the respiration became fuller and quicker; the lips resumed their colour; animation returned to the countenance; the uterus contracted powerfully, and expelled several coagula; in short, the patient returned to life, and did well.

"Did the percussion of the effort to vomit stimulate the uterus to contraction? Would it be safe practice, in cases of uterine hæmorrhage, to excite vomiting to obtain this end? It is a most powerful mean of bringing on re-action, as we often see in performing venesection; for the patient being faint, the stream of blood faulters; but no sooner is vomiting excited, than the colour returns to the pallid cheeks, and the blood, (almost as florid as arterial) rushes impetuously from the orifice. This determination to the surface may relieve the interior of any undue onus, and, therefore, prove of service in internal hæmorrhage. I once saw a case of hæmoptysis, which proceeded to an alarming extent, yield to small doses of sulphate of magnesia, which, from a peculiarity of the patient, invariably excited vomiting. The hæmorrhage did not return after the second dose. Why, in syncope, should the venous blood become florid?"

### *Widows' Fund of Medical Officers.*

On the 16th of April and 26th of May, the Navy and Army Medical Officers celebrated the Anniversaries of their respective Societies for the benefit of Widows; the *former* at the Free Mason's Tavern, Commissioner SEARLE in the Chair—the *latter*, at the Thatched House Tavern, GEORGE DICKSON, Esq. Chairman.

At these two meetings many distinguished characters, Military, Civil, and Medical, were present, and the greatest harmony, conviviality and decorum prevailed. It is gratifying to our best feelings, and honourable to human nature itself, to see our military and naval brethren of the profession, after a long and hazardous service in mitigating the horrors of war, by pouring balm into the wounds of friends and foes, unite, in the leisure of peace, to contribute, from the scanty pittance of their half pay, towards a fund of relief for those who shall survive them, in this sublunary existence. We had the happiness of witnessing the enthusiasm, in the cause of philanthropy which manifested itself at both these Anniversaries —a philanthropy which will perpetuate the eternal honour of these two corps, and stimulate others to imitate their bright example.

These social annual meetings of men who, for twenty years, had been exposed to the dangers of "flood and field," are calculated to call up a host of pleasing reflections on the toils which they had endured, the hazards they had escaped, and the victories which they had witnessed of their country's arms. Who among them did not verify the prediction of Æneas?

"Forsan et hæc olim meminisse juvabit!"—

especially when he reflected on the provision which he was then voluntarily making for the disconsolate widow and orphan—a provision which, small as it is, makes all the difference between absolute want, and comparative comfort, in the situation of those for whom it is intended!

After the toasts, songs, and sentiments had taken their merry rounds, and the sons of Mars and Neptune—

"Had toom'd the stoups to friendship's growth,
And days of Lang Syne."

The company broke up, and retired highly pleased with the excellent arrangements of the Stewards, and the dignified conduct of the Presidents.

---

*Hunterian Society.* The first Annual Meeting of this Society was held at the King's Head in the Poultry, on the 10th of February, Sir W. BLIZARD in the Chair.

From the Report made by the Secretary, it appeared that there had been a considerable accession of Ordinary and Corresponding Members; that many interesting communications had been made to the Society; and that the balance in the Treasurer's hand amounted to *£.* 66 : 4*s.* : 11*d.*

Liberal Donations of Books have been received in the course of the year from Dr. Johnson, Dr. Uwins, Dr. Nicholls, Sir W. Blizard, and others.

The following Gentlemen are elected Officers for the ensuing year:

PRESIDENT, Sir W. BLIZARD, F. R. S.

VICE PRESIDENTS, JAMES HAMILTON, M. D.; JOHN MEYER, M. D.; BENJAMIN ROBINSON, M. D.; LEWIS LEESE, Esq.

TREASURER, BENJAMIN ROBINSON, M. D.

SECRETARIES, JOHN T. CONQUEST, M. D. F. L. S.; W. COOKE, Esq.

COUNCIL.—Thomas Bell, Esq. F. L. S.; Isaac Buxton, M. D.; Thomas Callaway, Esq.; W. D. Cordell, Esq.; Robley Dunglison, Esq.; John Dunston, Esq.; Henry Greenwood, Esq.; William Kingden, Esq.; Charles Meeres, Esq.; James Miles, Esq; Benjamin Pierce, M. D.; Henry Richard Salman, Esq.

Communications addressed to the Secretaries, No. 10, St. Mary Axe, will be thankfully received.

*Poisoning by Verdegrease.* A lime-maker having dined off some peas which had remained, since the preceding day, in a copper vessel, was seized with violent pains in the stomach and belly, accompanied by vomiting and purging, with tenesmus. Milk and olive oil had been given by the attendants, before the physician arrived, but with the effect of increasing the violence of the symptoms. Warm water and gum were exhibited by the order of Dr. Saurel; but the vomitings and spasms continued unabated. A mixture was now administered, composed of sugar, water, whites of eggs, and powdered charcoal. The vomitings ceased, and a second draught removed entirely the epigastric pains. The patient fell asleep, and awoke quite well. *Journal Gen. de Medicine.*

Mr. Marks, of Stapleton Place, near Bristol, was lately successful in a case of tetanus, by means of large bleedings, cupping near the spine, and the daily use of purgatives so as to procure four or five motions daily. Eighty ounces of blood were abstracted, and the *third* bleeding shewed the inflammatory buff. Mr. Baynton saw the patient, and suggested the above mode of treatment.

*Academical Institution.* Mr. William Scott has founded an Academical Institution, (somewhat on the plan recommended long ago by Milton, in his Plan of Education) in Lothian Street, near the College, Edinburgh, more particularly intended for Students in Law and in Medicine. We have been favoured with a Prospectus of this Institution, and beg to draw the attention of medical students in Edinburgh to it, as it promises to be of great utility.

*Neuralgia Fascialis.* A thin, robust man, 53 years of age, in the habit of being exposed to all the vicissitudes of the weather, received a blow on the right side of the head, which felled him to the ground, and rendered him insensible for some minutes. This was followed by some headache; but, in the course of two months, a fascial neuralgia was developed, of the most intolerable violence.

---

In the Press, and shortly will be Published, Underwood's Improved Catalogue of Books, in Anatomy, Medicine, Surgery, Midwifery, Chemistry, Botany, Materia Medica, Veterinary Art, &c.; with a Table of Contents methodically arranged: to which are added, Tables of the Pay of the Medical Department of the Army, Navy, and East India Company's Service; a complete List of Lectures delivered in London, &c &c.

Dr. A. P. Wilson Philip has in the Press, in One Vol. 8vo. a New Edition of his Treatise on Symptomatic Fevers; which, with the New Edition of his Treatise on Simple and Eruptive Fevers, just Published, will comprehend all Fevers and all Diseases attended with Fever.

Dr. J. Gordon Smith, Lecturer on Medical Jurisprudence, is preparing for publication a work on that subject; in which particular reference will be made to the examination of medical evidences, touching those matters on which they are required to give professional testimony in the British courts. It will also be intended as a Text Book to his Lectures; and is expected to be ready early in next season.

---

### ANALYTICAL SERIES.

Most of the back Numbers of this Journal being scarce or out of print, and the Editor not being able to superintend the republication of so many Numbers, a New Series is commenced, and such an impression thrown off as is not likely to be soon exhausted. This measure produces no inconvenience to those who hold the Quarterly Series; while to new Subscribers it affords a point of starting with a complete work, and without any expence of purchasing preceding Numbers.

Although the nature and arrangement of the Journal are incompatible with distinct original communications, the Managing Editor will thankfully receive practical hints, observations, or strictures on recent works or prevailing doctrines, modes of practice, &c. &c. for the good of medical science in general. Where it can be done with propriety or consistency, the sources of information will be publicly acknowledged. All communications will speedily reach the Editor, wherever he may be at the time, if addressed as directed in the title page.

### WORKS RECEIVED FOR REVIEW.

1. A TREATISE on the Diseases of the URETHRA, VESICA URINARIA, PROSTATE GLAND, and RECTUM. By Charles Bell, Surgeon to the Middlesex Hospital, and Lecturer on Anatomy in the School of Great Windmill-street. *New Edition*, with Notes, &c. &c. &c. By John Shaw, Surgeon; Demonstrator of Anatomy in the School of Great Windmill-street. Octavo, pp. 416. London, 1820.

2. Des PRISONS telles qu'elles sont; et telles qu'elles devraient etre. Par Louis René Villermé, M. D. &c. Paris, 1820. Octavo, pp. 192.

3. Observations on Strictures of the Rectum, and other Affections which diminish the Capacity of the Intestine: including Spasmodic Constriction of the Anus, the Hæmorrhoidal Tumours, Excrescences, and the Prolapsus Ani; and the Mode of Treatment, accompanied by Cases and Engravings. By W. White, one of the Surgeons to the City of Bath Infirmary and Dispensary. Octavo, 1820. *Third Edition, improved.*

4. Revue Médicale, Historique et Philosophique. No. 1 and 11, January and March, 1820. Octavo. Paris.

5. MEMOIRE sur l'Utilite des PIECES d'ANATOMIE ARTIFICIELLE Chirurgicale. Par J. F. Ameline, Chirurgien, Professeur d'Anatomie a Caen, &c. Octavo. Paris, 1819.

6. Practical Observations on the Medical Powers of Mineral Waters, and of the various Modes of Bathing, &c. &c. By Patrick Mackenzie, M. D. Licentiate of the Royal College of Physicians, and Assistant Physician to the Fever Institution. *Second Edition, enlarged.* Octavo. London, 1820.

7. The American Medical Recorder of Original Papers and Intelligence in Medicine and Surgery. Conducted by John Eberle, M. D. &c. &c. of Philadelphia. Vol. 1. Philadelphia, 1819.

8. Letters to the President of the Associated Apothecaries and Surgeon Apothecaries of England and Wales, on the present state of the Practice of Physic and Surgery, *first Series*; intended to give a comparative view of particular systems of medical education; to consider the separation of medicine from surgery; to estimate the claims of the general practitioner; and to propose a more respectable mode of remunerating his attendance. Octavo, sewed, pp. 77. London, 1820.

9. An Inquiry into certain Errors relative to Insanity; and their Consequences; Physical, Moral, and Civil. By George Man Burrows, M. D. F. L. S. &c. &c. Octavo, pp. 320. London, 1820.

10. Memoire sur les Fungus Medullaire et Hæmatode. Par J. P. Maunoir de Genève, Professeur, &c. &c. 1 Vol. Octavo, pp. 137. Paris, 1820.

P. S. The above Memoire gained the Prize of 400 Francs, offered by the Royal Society of Medicine, of Bourdeaux, for the best dissertation on the subject.

11. An Elucidation and Extension of the Harringtonian System of Chemistry, explaining all the Phenomena, without one single Anomaly. By Robert Harrington, M. D. Author of "Fire and Planetary Life." Octavo, pp. 170. London, 1819.

12. Practical Observations on the Symptoms, Discrimination, and Treatment, of some of the most common Diseases of the lower Intestines, and Anus; particularly including those Affections produced by Stricture, Ulceration, and Tumour, within the Cavity of the Rectum: and Piles, Fistulæ, and Excrescences, formed at its external Opening. Illustrated by Cases, &c. &c. By John Howship, Member of the Royal College of Surgeons, &c. &c. &c. One Volume, Octavo, pp. 176. London, 1820.

